# What, why and how? Meta-Reflections on Cultural Psychological Approaches to the Scientific Study of Phenomena Called Religious

**DOI:** 10.1007/s12124-018-9427-9

**Published:** 2018-05-09

**Authors:** Jacob A. Belzen

**Affiliations:** University of Amsterdam, Amsterdam, Netherlands

**Keywords:** Cultural psychology, Interdisciplinarity, Religion, Essay, Modesty

## Abstract

This deliberately essayistic paper deals with strengths of and limits to cultural psychology, especially in its application to research on religion. It is presented as only one possible approach, composite in itself and drawing on a variety of theories, insights, methods and techniques, but working on one of the fundamental aspects of human psychological functioning, and therefore as indispensable to efforts to explore and understand anything called religious as any other psychological approach may be. Furthermore, the paper makes an explicit plea for an interdisciplinary approach to psychology. Whether researchers will employ cultural psychology or another approaches from contemporary psychological sciences will depend on their personal preferences, their professional training, the type of context they are functioning and hopefully also on the kind of phenomenon pointed out to them as religious by a certain (sub)culture.

## An Essayistic Approach

The task set before us by the organizers of the present special issue is important, and one that receives too little attention from psychologists absorbed in the demanding business of their empirical research. The task requires to take a step back from the daily, all too ordinary occupations and urges the participants to reflect on why they are doing what they think (or even claim) they are doing. The request is, in my reading, not for a presentation of empirical research, not for discussion or evaluation of data, conclusions and implications, but for an exposé of the reasons for employing this or that approach in psychological research on religion; the request is not even primarily for a presentation of methods and techniques, but for an exercise in methodology, for fundamental reflection on such methods. The request is also a challenge to answer the questions raised in a personal manner, thereby allowing for a somewhat essayistic style – “essay” understood according to the French, not to the Anglo-Saxon tradition.

In general, an inquiry into these issues is of utmost importance as it is about the very foundations and premises that are guiding the course of empirical research, sometimes even without researchers being aware of it. From the history of science at large, abundant examples are known from entire research traditions that have been abandoned, not because the many pieces of empirical research actually carried out within that tradition were wrong, but because other foundations for dealing with the same kind of questions came into being, or because other apriori’s took lead in the development of a certain field. An enormous literature on scientific revolutions, on paradigm-shifts, on progressive versus degenerative research programs deals with these issues, a literature that is becoming increasingly empirical itself, as next to philosophical questions about rationality the actual course of research is taken into account (Feyerabend 1975/2010; Kuhn 1962; Lakatos 1978; Latour 1987). It testifies to scientific research being a very human, even all-too-human (Nietzsche 1878) activity, which is certainly not only driven by a serene search for truth. While sociological perspectives are commonly integrated in historical and philosophical research on the course of the development of that “thing called science” (Chalmers 1976/2013), psychological perspectives are seldom brought to bear. A publication like *Psychology of science* (van Strien 2011) is a rare one, but while highly readable and recommendable, the book draws far more on other disciplines that on psychology. Next to “creativity”, its author introduces “the personal factor” in his striving to analyze psychologically, but the treatment is mainly by way of anecdotes. Yet, to psychologists it should be self-evident that in all human activities very personal, biographically determined factors are at work. A by now in academic psychological circles no longer very popular approach like psychoanalysis focuses precisely on such factors.

Psychology of religion is no exception to all of this. Here too, the choice what to do research on and the choice for the way in which to proceed is determined by a number of very different, yet highly influential factors. Let us briefly distinguish three clusters. (1) Personal, biographical, perhaps even psychodynamic factors are usually relevant among the reasons why someone involves in research on religion. While it might be illuminative, this paper will not continue into the direction of exploration of this “context of discovery” (Popper 1934/1959). It would require in-depth treatment of a number of case-studies (like Belzen 1991), which is beyond present possibilities. (2) To a second cluster of factors I will refer only briefly as well, as they will be almost self-evident, namely the multiple contexts of the researchers. (2a) The choice for the type of psychology to employ in research on religion will depend on the type of psychology a researcher has got acquainted with at all during her education and training as a psychologist, on the kind of employment she envisioned for herself and on the kind of specialization she acquired (see also Belzen 2015). In all likelihood, someone trained as an experimental cognitive psychologist will proceed differently from someone who qualified as a psychotherapist. (2b) Next to education and training, the context of functioning will be highly influential in the choice of approaches, methods and techniques as well: a psychologist employed at a Max Planck institute will proceed differently from someone teaching students in a ph.d.-program in social psychology. (3) The cluster receiving at least some attention, perhaps particularly in the psychology of religion, is the ensemble of apriori’s and theoretical assumptions guiding research or professional activity. Psychologists try to find out more about something which they have a notion of, but no precise knowledge about: they want to enlarge our insight in human psychological functioning per se, or about this psychological functioning in a number of empirical constellations (ranging from personal experiences to structures in society). As is always the case, however – so this remark is not a criticism, but an observation – preliminary notions, often enough not reflected, play an important role, as is pointed out by philosophers of psychology, theoretical psychologists and perhaps others more. I said that with regard to psychology of religion the reflection on such assumptions is perhaps more elaborate than with regard to other fields of psychological inquiry, as sometimes people with considerable training in precisely detecting and analyzing such foundations, like philosophers and theologians, also reflect on the psychology of religion (Henning 2000; Henning and Nestler 1998; Klünker 1985; Machon 2005; Nørager 1996a).

As may be clear, these three clusters are not independent. The kind of apriori’s with regard to religion (so, cluster c) will depend highly on biographical factors (a) and will be understandable from factors like education and training (b) as well. However, things tend to a bit more complicated perhaps in the case of psychology of religion than in other fields of psychological research as often enough researchers, more or less conscious, set out to *prove* the truth of the assumptions they adhere to. They set out to see and find what they assume to be the case. While to some extent, this is how all human perception functions, in the case of the psychology of religion it may lead to questionable results. A virtually famous case from the early decades, now largely forgotten, would be Carl Girgensohn (1875–1925), a student to legendary Külpe and Bühler, who was one of the very first to apply the experimental psychology of those days – a procedure very different from the present one! – to the study of religion. His study *The psychological structure of religious experience* (1921/1930 immediately turned him into one of the leaders of the psychology of religion in Europe: the study was monumental, after working for some 15 years on it, the publication became a book of some 700 pages. It had taken some courage to undertake a study of such a delicate topic as religion with the help of so mundane an approach as “experimental psychology”. Girgensohn received personal congratulations from many leading psychologists, he became a member of a number of psychological associations, was invited to preside the plenary session on psychology of religion at the 8th International Congress of Psychology, but died suddenly in 1925. Many decades later, theologians pointed out, however, that Girgensohn established in his elaborate work what he had thought all along: in 1903 he defended already the thesis, against the popularized reading of Schleiermacher, that religion would primarily not be anything emotional, but a psychological “I-function”, central to which would be the concept of God (Henning and Murrmann-Kahl 1998, p. 100).

An example from more recent psychology of religion would be the claim, presented by psychologist of personality Ralph Piedmont (1999), to have shown that the so-called Five-Factor Model of Personality (FFMP) needs to be enlarged in order to include “spirituality” as the sixth factor of personality. This claim is consistent with a dominant tradition in classical Christian theology, rooted in the works of St. Augustine and other so-called Church Fathers, who taught that a desire, or an orientation towards “God”, is intrinsic to the human being, who would be religious by nature, so to say, and who would only be at rest, “whole”, fully human or phrased in what positive way ever, if living in accordance with divinely-eternal destination. In reaction to critical ideas about religion since the European Enlightenment, this idea has been voiced in numerous, usually apologetic philosophies, defending the existence of religion, and claiming that being religious is a kind of default setting to humans. Next to personal reasons to defend religion, it has led in the psychology of religion to a major stream of clinical research, asserting that religion is or at least can be beneficial to mental health.

It is important to note that the existence of such apriori’s need not necessarily disqualify the research that is based on it! The concept of the psychology of religion having been employed most frequently in empirical research, has been the typology of religious attitudes as initially proposed by Allport, who distinguished between intrinsic and extrinsic religiosity. Allport had been upset when empirical research repeatedly showed that all kinds of vices, like prejudice, were more prominent with believers than with non-believers. The introduction of the typology named restored peace of mind: the vices would be found primarily with extrinsically religious people, far less with intrinsically religious ones (Allport and Ross 1967).^1^ But as said, Allport’s personal religious sentiments do not disqualify the distinction between intrinsic and extrinsic religiosity on the level of psychological attitudes. One might well become suspicious, however, when running into much further reaching apologetic implications presenting intrinsic religiosity as correlated with what a certain researcher considers to be metaphysically true religion or to be clinically healthy religion.

These few examples should suffice to remind us that there is ample reason to inquire, as our task in this special issue is, into the reasons why researchers turn to what they present as religion and into the way they construct their objects of religion and their procedures in empirical research. On closer scrutiny, so it seems, it is far from clear what all the terms employed in the psychology of religion really signify. As we will see in a moment, “psychology of religion” does not mean the same to everyone, but neither does any of its constitutive terms! Not only are the terms “psychology” and “religion” very differently understood by different researchers, the initial question as to “why” to study religion could evoke answers and reflections as embracing and lengthy as entire books. In an effort to stay within manageable portions of text allowed, the present paper will not enter a discussion of the reasons one might have to get involved in the psychology of religion at all, fascinating as such a topic as such without doubt would be. I shall spend a word on some of the conditions of work in the psychology of religion somewhat later, however, as the reasons for engaging in such research at all may be highly influential in the way psychologists perform their research.

The general stand that is at the basis of my exposé will be one of sincere modesty. It is my conviction that not only in the psychology of religion we are dealing with issues of high complexity, which despite all our hard scientific work we hardly understand appropriately, and that in our daily work on contributing to any scientific enterprise we all too readily forget that we are only adding – if we are adding anything at all! – a little stone of a certain kind to a much larger mosaic that the ensemble of our field is. At best, the kind of piece we are working on is necessary and indispensable, yet it will only be one stone within a larger whole. And even the entire field we call psychology of religion is just an element of much larger and more complex ones. Leaving aside perspectives from *Religionswissenschaft* or even Theology, and restricting ourselves to psychology, let us remember that the field between the poles “psychology” and “religion” is huge and complex, “psychology of religion” being just a very minor part of it.

## Psychology of Religion?

While many Westerners may think they know what the words “psychology” as well as “religion” signify, it is different with the phrase “psychology of religion”: most people, professional psychologists being no exception, have never heard about it. The most problematic element is probably the little word “of” in the designation “psychology of religion”: what does that “of” stand for? Briefly said, it does not mean a psychology that would belong to any religion. One certainly could conceive of something like that: a “religion” – if it is a religion! everything depends on definitions here, but we will come to that later – like Buddhism is very psychological in nature, it talks about psychological phenomena and processes, and its inherent “psychology” – quite different from the “psychology” as developed in Western universities and research centers – can be spelled out and confronted (or maybe reconciled) with other types of psychology. Interesting at that may be, hardly any Western psychologist would accept such an enterprise as a specimen of psychology. (Whereas Western psychologists would regard it to be a kind of philosophy, or a branch of some cross-cultural scholarship, Buddhist scholars would hardly be interested in any comparison with Western psychology, as to their understanding the latter is rather trivial, not involved in the pursuit of wisdom as they are doing.)

In the designation *psychology of religion* the “of” therefore does not mean not something like “property of”, but rather “about”: psychology of religion is a Western academic enterprise, employing Western psychology, dealing with “religion”. And here we should immediately differentiate further, for employing psychology is not reserved for psychologists, of course. Any scholar can read and bring psychological theory into a dialogue about anything. (Whether this always leads to valuable reflections, is a totally different question.) In general and remarkably enough, one can probably say that the employment of psychology in dealing in a theoretical way with religion is often done by others than psychologists; this kind of work is usually called ‟psychology *and* religion”, sometimes bringing psychology and religion into some kind of dialogue, sometimes employing present day psychology (oftentimes a type of psychoanalysis, or Jungian theory) to interpret classic religious texts (not only “holy” scriptures, but also great religious thinkers like Augustine, Calvin or Kierkegaard, see e.g. Dixon 1999; Parsons 2013; Westerink 2012). Distinct from this, the psychological dealing with ‟religion” (perhaps better circumscribed as: dealing with “some contemporary behavior considered religious in a particular context”) in an empirical way, whether it be in a piece of research or in some kind of health treatment, is usually done by psychologists.

Even if we restrict the understanding of the phrase “psychology of religion” to empirical dealing with religion by persons trained and formally qualified as psychologists, we need to be aware that the major part of that empirical work is usually not called “psychology of religion”. For decades and perhaps still at present, psychologists mainly found employment in segments of society referred to by labels including words such as clinical, health or health care, therapy, counseling and so forth. This practical work with patients, or in general with clients applying for help to a psychologist could logically be called psychology of religion. But it isn’t! The phrase “psychology of religion” usually refers to psychological research on religion only (Hood et al. 2009; Miller 2012; Paloutzian and Park 2013).^2^ It is this kind of work that one finds reviews of in the increasing number of present day “handbooks”, introductions and other overviews of the psychology of religion, typically authored by psychologists and published by psychology presses. Although one does find in the psychology of religion-literature a considerable number books and articles on religion in clinical psychological practice (Griffith 2010; Huguelet and Koenig 2009), it is the research on the role of religion in health and/or illness and the research on how psychologists perhaps are handling religion in mental health care or psychotherapy that goes as psychology of religion, not the practical work by psychologists. (So, illogical as it may be, this means that most of the professional dealing with religion by psychologists is not included in the common understanding of the phrase. Only very recently the American Psychological Association launched a journal *Spirituality in Clinical Practice,* but even there, one mostly finds research reports.)

Let us take just one more differentiation into account: just as we noted already that, in principle, a non-psychologist may read psychological literature (perhaps even ending up knowing more about psychological theory than many formally qualified psychologists), it is, in principle, possible for other scientists than psychologists to employ research methods and techniques developed or commonly used by psychologists. Practical theologians, for instance, often are interested in psychology and some of them engage in some kind of psychology-like research (e.g., Fowler et al. 2004). Indeed, there exists an entire field called “pastoral psychology”, that closely resembles psychology of religion, yet must be distinguished from it (Watts et al. 2002). The main difference is the intention of pastoral psychology: as it very name suggests, it aims to serve religious purposes, usually those of some Christian church. Pastoral psychology is psychology – whether performed by a psychologist or a psychologically trained theologian – in the service of pastoral work, whether in an applied form like counseling or anything that might help to make the pastor function better, or in the form of empirical research, investigating the ways in which the Church may be facilitated. (“Psychology and religion” as well as “psychology of religion” formally stay neutral with regard to the type of religion they deal with, although it probably has to be admitted that especially in the psychology of religion considerably sympathy for Christianity is at work.)

## Pluralism Squared

Laudable in itself as the effort to be precise in the understanding and employment of the term *psychology of religion* may be, the lack of clarity about each of the constitutive terms is a far bigger problem. For what is psychology? What is it about, or supposed to be about? And what does the term “religion” mean?

Perhaps amazing to psychologists, the theoretical confusion with regard to the term “religion” is certainly not smaller than that with regard to “psychology”: it is not clear at all what religion is or what the term means. Or even stronger formulated: what counts as religion to one author, may count as a perversion or as anything-but-religion to others (the religious phenomenon called “temple prostitution” may be abhorred by advocates of religious “celibacy”; the phenomenon called “religious terrorism” by some, will be called “martyrdom” by others, to give just some examples of highly controversial practices and labeling). It seems that the word “religion” refers to something different in different contexts and in different discourses, and in the literature dealing with its definition, it is increasingly pointed out that the noun “religion” is unsuitable to refer to the worldwide multitude of phenomena that Westerners have called by that name. Indeed, recently, voices can be heard that propose to discontinue altogether the use the word “religion” in scholarly discussions: the very word would be coined by Westerners, modeled after a particular understanding of certain types of Christianity and in its application to utterly different subcultures it would be an example of intellectual colonialism (Feil 1986, 1997; Haußig 1999). The diversity supposedly covered by the noun “religion” as its designation would have only in common that it is being referred to as religion (by Westerners), the real issue for scholarly research being to find out, why some practices at some time have come to be regarded as “religion” at all (see, e.g., Hölscher 1999; McCutcheon 2007; Taves 2009). In the empirical sciences of religion (primarily among them history, anthropology, sociology and psychology) this is reflected in a great diversity of references to the object of a particular investigation: some people meant by “religion” Christianity or one of its denominations (taking, e.g. Roman Catholicism as a model of all of Christianity or of all religions), but some have meant all-other-religions-than-Christianity (usually implying Christianity being right, the other ones being false); speaking about religion, some researchers were only investigating religious experiences (and among these often only conversion experiences). Also trendy fashions have reflected themselves in the way research was conducted: numerous publications have taken church membership as operationalization of religiosity, as if there were no religiosity outside of churches; nowadays, the opposite trend is growing: by employing the label “spirituality” also individuals who claim to be religious but not a member of any religious group should be included into research samples. Conceptual problems and especially overgeneralizations like these have been haunting all empirical sciences of religion since their inceptions. Only a modest stand will present a way out, also for the psychology of religion: instead of claiming to have found out facts about *all* religion, about all religiosity, about all religious experiences, etc., it will be more honest and appropriate to admit that a piece of research only found out something with regard to a specific phenomenon found with a specific sample in a specific context, only with great caution to be generalized to a population that *may* be found in other contexts as well.

For if anything is clear about “religion”, it is that the word is used to refer to an almost endless variety of empirical phenomena, different from culture to culture (sometimes even from contexts to contexts within a single culture), different from period to period within the same culture, and sometimes even different among the adherents of the same religious tradition at the same time and place in the same culture. (And psychologists, with their traditionally great attention to the individual, are prone to add: even in the life span of a single person religious experiences will differ: a religious conversion at the age of 17, for example, is likely to be psychologically differently structured than a religious conversion at the age of 71.) In conclusion and as a pragmatic solution to the problems about the conceptualization and definition of religion: psychology needs not itself define the concept of religion (such can logically be left to academic disciplines like philosophy and comparative religion), a psychologist can, however, turn to a specific phenomenon that, within a specific culture, is (generally) considered to be religious, and start doing research. Having done a sound piece of research, it can next be established to what extent its results apply in other contexts as well.

Contrary to what outsiders to the field may assume, among psychologists there is not much consensus about what psychology is either. Sometimes there exist veritable wars among different fractions of this heterogeneous field, the by now classic example being the stance taken towards psychoanalysis: whereas many lay persons, when hearing the word “psychologist” still think of a psychoanalyst listening to patients who are lying on a couch, to many professionals this school of theory and practice doesn’t even count as psychology, not even as a part of science at all. “Psychology”, whatever it may be, has become an enterprise in which so many people are involved and within which so much is going on, that representatives from different corners of the field oftentimes are no longer aware of what is going on in other parts. To some extent, this is the price all scientists have to pay for their increasing specialization, and it is an epiphenomenon to the ever increasing number of people involved. But things are a bit more complicated when it comes to psychology, which seems to be a science about an evasive object. Although hardly anyone is still using the word “psyche” anymore, the phenomena that belong to the psychic realm seem to be clear enough. The sense that people have of being a self, or of having an identity, may count as an example of a psychological phenomenon, of something of a nature all of its own, requiring a special, independent science to conceptualize and investigate it. Activities we commonly call “thinking”, “dreaming”, “desiring” would be other examples. To many, even today, it seems that psychological phenomena are something internal, something within human people, something invisible to others, but accessible to the subjects themselves: the subjects themselves would know their thoughts, desires and feelings. Only, what this “internal”, “within” would mean has never been clarified: clearly it does not mean within bodies, for within bodies we find organs, blood and other liquids, but no thoughts or aspirations. From the history of psychology we know that the problems in conceptualizing the psychic realm have been so large that to leading psychologists so-called behaviorism for some time seemed to a solution to the perennial difficulty of defining psychology and its object: instead of exploring anything “internal”, behaviorism stated psychology should study something “external”, namely human behavior. Only, behavior is not an object of inquiry specific to psychology at all: a great number of disciplines (history, economics, sociology, anthropology and more) study human behavior too, but asking different questions than psychology. What is or should be specific about the psychological study of human behavior (as material object) is the formal object which is given with the existence of psychic reality, however conceptualized. And to be even more precise, psychology does not study any “behavior” as such, but it studies human acts, it studies what a specific act means to the actor, it asks for the motives involved, it asks, again, for realities seemingly somewhere “within” the actor.

Indeed, the efforts to conceptualize psychic reality are numerous, and although the supporters of this or that conceptualization oftentimes quarrel with one another, representatives of fields like theoretical psychology have tried to come up with accounts, at a somewhat more abstract level, to peacefully integrate the many different schools and approaches within psychology. The best known of these efforts, a bit dated now, is probably the one that presents psychology as a multiple science, belonging to and drawing on different sciences or scientific traditions. Psychology would be both a *Naturwissenschaft* and a *Geisteswissenschaft*, it belongs to both the natural sciences and to the humanities, as its very object, the “psyche”, bears the characteristics of the object of the natural sciences as well as of the object of the humanities. According to this classic distinction the natural sciences study material reality (like, e.g., stones or their composing elements), and the human sciences study cultural, *geistige* products of human activities in the cultural realm (e.g. poetry: a poem is cultural phenomenon, independent of its being printed or recited). Natural sciences study objects that hardly ever change and that remain the same for at least millions of years, human sciences study objects that are ever changing, that are particular to a certain time or that even have change as an essential characteristic (e.g. politics). The object of psychology belongs to both realms: identity, as a process that belongs to psychological functioning, cannot exist without the material foundation that is given with human corporeal reality. But identity is no corporeal reality, not even an epiphenomena to it: identity is a typical human phenomenon, given with humans being aware of their past and being able to project their future, which they do differently at different ages. Humans in general do not only belong to the realm the natural sciences do research on, humans are cultural-historical beings that cannot be conceived of without taking their being culturally into account. (And therefore, the anthropological movement in medicine likewise pointed out that also the human body exceeds an ensemble of physiological functions.) Although nothing psychological – attitudes, personality, volition, emotion and what have you – can exist without a brain, to think or to will or to feel and what have you cannot be reduced to brain activity only. Elements of psychic functioning, like cognition, self or desire, are a historical reality, not only occurring at a certain point in time, but also characterized by their changing throughout the course of an individual life. They resemble and can be studied like texts, and psychology can fruitfully draw on other humanities for inspiration and models. Moreover, as a number of psychological thinkers as different as Vygotsky, Mead, Lacan and many others have pointed out, the elements of psychological functioning as commonly studied in Western psychology only come into existence due to the human neonates being inculturated. (So, not only which language one speaks will depend on the subculture into which one is born, the point is more radical: speaking as such only occurs because of being spoken to, because of being treated as a (future) member of a given culture. No attitude, no self, no meaning or anything psychological comes about just by itself, it all co-depends on inculturation.)

At present, there does not seem to be much dispute about this anymore. Usually, the classic dualism hinted at is enlarged to a tripartite structure distinguishing at least three fundamental aspects of psychic functioning: the neurological, the developmental-longitudinal (oftentimes including the psychodynamic viewpoints) and the social (or systemic) – aspects that cannot be reduced to one another, but that in every single case interact specifically. Based on each of these fundamental dimensions of human psychological functioning, a large variety of (sometimes small-scale) theories has been developed that is applied in the exploration of countless phenomena: traffic, music, love, bereavement, health behavior, politics and what have you can be studied under the aspect of this multiple psychological perspective (as all of them could be studied under that aspect of many other sciences: history, economics, law, etc.) as well. It has led to a situation in which it may be more appropriate to speak no longer about “the psychology of x”, but about “the psychologies of x”, explicitly acknowledging the multiple and usually not integrated character of the many types of psychologies existing today.

If we turn to religion as a possible object for research by psychologists, we should recognize that this leads a kind of multiplication of two plural entities: religion, as pointed out above, is multifarious, and so is psychology. Therefore, it may be better to speak, more modest, about “psychologies of religions” than about “the” psychology of religion. (Or, to be even more precise and in any case more neutral, about “psychologies of phenomena – whether events, experiences, behavior or states of affairs – called religious at a certain point in time”, for, as pointed out, what some call religious, is no religion according to others.) That there is not much rectilinear progress in psychology (whether “of religion” or of any other field), as is lamented sometimes by some authors, calling for integration of scattered knowledge and insights (e.g. Hood et al. 2009; Spilka and Ladd 2013), should not come as an amazement, but as a logical consequence of the scientific discipline of psychology being structured the way it is. And what we might call “cultural psychology” is also just a part of psychology, albeit an indispensable part.

## On Cultural Psychology

Though clearly in favor of cultural approaches in the psychology of religion (Belzen 2010), my perspective on cultural psychology is a modest one too. Contrary to what sometimes is suggested, cultural psychology is no new development. In fact, what is called cultural psychology is heir to number of classical, mostly continental positions that did not isolate persons and world, but rather understood persons as beings-in-the-world, a perspective also philosophically articulated by phenomenology as exemplified in the work of Heidegger (1927/1972), Merleau-Ponty (1945/1962), Ricoeur (1981) and many others. Like psychology in general, contemporary cultural psychology is a rather broad, heterogeneous enterprise, to which many well-known psychologists have made significant contributions. It is important to realize from the onset that cultural psychology is not a psychology entirely different from other kinds of psychology as developed during the discipline’s past, nor is it one of its separate subdisciplines or simply a field of application. Broadly stated, and at this point without much specification, cultural psychology is an approach within psychology that is trying to describe, to investigate and to interpret the interrelatedness of culture and human psychic functioning. It is the part of psychology that tries to take serious the perhaps seemingly trivial observation that both of these would not exist without one another, and that therefore culture is a major factor in all meaningful human conduct and on the other hand traces of human involvement can be traced in all expressions of culture. Culture is here understood as a system of signs, rules, symbols and practices that on the one hand structures the human realm of action, and on the other hand is being (re)constructed and transformed by human action and praxis. It is because religion is an element of human culture, that human beings can be religious.

It may be instructive to divide cultural psychology at large in different variants (that are obviously not entirely independent from one another, and that cannot all be dealt with in depth in this paper).

- First of all, and vital to the development of psychology as a body of knowledge, attitudes and skills, cultural psychology investigates how culture constitutes, facilitates and regulates human subjectivity and its expression in diverse psychic functions and processes as postulated and conceptualized by different psychological schools and theories (e.g. perception, memory, mental health, the self, the unconscious, etc.). It is important to note, that the concept of culture employed here is a dynamic one, it does not just mean “context” or “situation”. In the words of Ernst Boesch, a major German representative of contemporary cultural psychology:

Culture is a field of action, whose contents range from objects made and used by human beings to institutions, ideas and myths. Being an action field, culture offers possibilities of, but by the same token stipulates conditions for, action; it circumscribes goals which can be reached by certain means, but establishes limits, too, for correct, possible and also deviant action. The relationship between the different material as well as ideational contents of the cultural field of action is a systemic one; i.e. transformations in one part of the system can have an impact in any other part. As an action field, culture not only includes and controls action, but is also continuously transformed by it; therefore, culture is as much a process as a structure (Boesch 1991, p. 29).

With such conception of culture, cultural psychology goes beyond the common understanding of culture in psychology at large. Whereas contemporary psychology generally recognizes that not only human interactions are influenced by culture, but that also individuals’ feeling, thinking, experiences and behavior are shaped by it, cultural psychology conceives of these as being inherently cultural: as being the result of human embeddedness in culture, which is therefore to be considered as a genuine element of all human functioning relevant for psychology.^3^ This form of cultural psychology will be dealt with at greater length in this paper. It is the form of cultural psychology usually developed by psychologists. (This latter remark should not surprise, for, as we shall see in a moment, there are also other academic disciplines that employ or even make contributions to psychology as a scientific enterprise.)

All conditions and determinants of psychic functioning, whether they are limitative (like psychophysical makeup or social and geographical conditions), operative (like acquired, learned activities), or normative (like rules and norms), are always cultural-historically variable (cf. Peeters 1994; Wagoner et al. 2015). Therefore, this first variant of cultural psychology consists, roughly, in two forms: a synchronic and a diachronic one. In both forms there is a realization of the historical nature of culture (in its various manifestations) and therefore of human psychic functioning. Yet, in the first form, the emphasis is on psychic functions and processes in contemporary subjects; there is an abstraction of historical variation. In the second form, however, the historical changes in human psychic functioning are being investigated and explained on the basis of modifications in cultural conditions and determinations. Cultural psychology as a whole is an interdisciplinary approach, as will be readily understood with this first of its variants: in both forms of the first variant distinguished here, cultural psychology is in need of collaboration with other disciplines from the social and human sciences. In the synchronic form, psychology relies on information, and sometimes theories, concepts and skills from disciplines like anthropology, sociology, political science. In the second one, historiography, and sometimes even evolutionary biology (Atran 2002, 2007), are among the obvious partners in theorizing and research.

(b) Secondly, numerous publications have traditionally been devoted to efforts to detect and determine the human involvement in all kinds of cultural products. Whereas in the first variant of cultural psychology, the understanding of culture is more or less anthropological, on a macro-level, in this second variant usually a much more elitist and restricted concept of culture is employed. Attention is given to products of so-called “high culture,” like novels, movies, operas and other arts, but also to entire areas like peace and war, sports, advertising, organizations, international affairs, and to important domains like socialization, sexuality and courting, labor, death and dying. Each of these subjects can and is also being studied by other scholarly discipline to which psychology in such case often relates as an auxiliary discipline. In fields (to be distinguished from disciplines!) like cultural studies, education or arts, the discipline of psychology is often called upon to explore the human involvement in the phenomena studied. In these cases typically some kind or another of psychology (very often: psychoanalysis) is applied. Although this may and has been done by psychologists (again: especially psychoanalysts) themselves, frequently in these cases, it is done by researchers and authors with an other than a psychological training. Or, if psychologists are hired in these contexts, they obviously are serving another goal than the development of (new) psychological theory.

In this second variant of cultural psychology, considerable attention has been given to a variety of religious phenomena, contributing substantially to the psychology of religion-literature. Not only numerous “great” psychologists, especially from the psychoanalytic tradition, have been writing explicitly on religion from the perspective of the psychological approach or theory developed by themselves (e.g., Freud, Jung, Erikson, Allport, Maslow, Fromm), but psychological approaches or theories have often been utilized by others to analyze some religious phenomenon. The latter has been done both by authors with a psycho(patho)logical training (e.g. Pruyser 1983; Kakar 1982, 1991; Meissner 1992, 1996; Rizzuto 1979), but frequently also by scholars with a (primary) background in theology, sciences of religion, or religious studies in general (e.g. Beth 1927, 1931a, b; Pfister 1910, 1926, 1944/1948; Sundén 1959/1966; Girgensohn, 1921; Holm 1990; Kripall 1995; Parsons 1999; Vergote 1978/1988, 1983/1996). As such work is covered at some length in other excellent reviews (as in Wulff 1997), this variant of cultural psychology will be left out of consideration in the remainder of this paper.

(c) A third variant of cultural psychology will be mentioned here even more briefly only. It is common to find an understanding among cultural psychologists that different cultural contexts, different times as well as different places, produce different psychologies, partly as a result of their being developed with/on subjects who are psychically differently constituted (cf. Gomperts 1992; Zeegers 1988), and that the history of psychology is not about natural facts, but about socially generated constructions (cf. Danziger 1990, 1997, 2008; Valsiner 2012). Therefore, within cultural psychology there is, on the one hand, attention to so-called indigenous psychologies: the psychologies as developed by “ordinary people” (as distinguished from Euro-American psychologists, who produced almost all of the present “academic” psychological knowledge), also in other parts of the world than on both sides of the Atlantic (e.g., Much 1995). On the other hand, there also is a fair amount of attention to the history of psychology as a Western enterprise. As will be clear, in this third variant there is again collaboration with experts on local cultures (whether academically trained in the Western tradition, like anthropologists, or not) respectively with historians, especially intellectual historians (or with historizing philosophers), cf. Belzen (1991, 2007), Laucken (1998), Paranjpe (1998).

Let us now turn to a closer exploration of the first variant just distinguished, to the form of cultural psychology concentrating on the cultural basis of human psychic functioning, developed as an integral part of academic psychology. After some very short general remarks, I will continue to deal with religion from the perspective of this first type of cultural psychology.

## Contemporary Cultural Psychology

As many cultural psychologists point out, it is important to distinguish between cross-cultural psychology and cultural psychology in a proper sense.^4^ The two disciplines work with different conceptions of culture, cross-cultural psychology operating with a rather traditional understanding of culture: it conceives of culture as a variable that may possibly take influence on behavior, and it investigates comparatively how experiences and behavior, attitudes, social relationships etc. present themselves within different cultural conditions. In its most straightforward form, individuals who match for age, sex, education and other relevant variables, but belong to different ethnic groups or live in different geographical regions, are being compared with regard to the psychic phenomenon the particular investigation focuses on. This type of research has contributed greatly to the present sensitivity for the cultural variations in human ways of experiencing and of being in general. Such comparative cultural studies often aim to determine culturally invariant forms of human expression, and considers these — in covariance with sociobiological perspectives — as anthropological constants, e.g. in research on emotions. In this approach, culture tends to be viewed merely as a qualification on the generality of psychological effects or as a moderator variable, but not as a constituent process that is implicated in explaining psychological phenomena (Billmann-Mahecha 2001).

Cultural psychology in a proper sense, on the contrary, stresses that cultural patterns of acting, thinking and experiencing are created, adopted and promulgated by a number of individuals jointly. Such patterns are supra-individual (social) rather than individual, and they are artefactual rather than natural. Therefore, psychological phenomena are cultural insofar as they are social artifacts, i.e., insofar as their content, mode of operation and dynamic relationships are (a) socially created and shared by a number of individuals, and (b) integrated with other social artifacts (Ratner 2002, p. 9). Conversion, e.g., is a phenomenon found within certain religions, having a different meaning within different subgroups of such religions, being the result of certain patterns of religious practice, in their turn related to certain religious doctrines and rituals. In cultural psychology usually the meaning of some form of action (or thought or experience) is central, not the action as such (which could be, and in fact, often is studied by other social and human sciences too). Culture, also cultural practices, is being conceived of as symbolic: it is considered to do more than merely represent preexisting realities and regulate behavior. Rather, culture is being seen as creating (social) reality, whose existence rests partly on such cultural definitions. With this, cultural psychology recognizes the open and indeterminate relationship between cultural meanings, practices and material forces. It is recognized that not only social institutions (e.g. marriage, school), roles (e.g. bride, student) and artifacts (e.g. wedding ring, lecture notes), but also psychological concepts (e.g. the self, emotion, mind) and epistemological categories (e.g. time) depend, in part, on cultural distinctions embodied in language categories, discourse, and everyday social practices.

The main contrast between both forms of psychology investigating the role of culture in psychological phenomena is therefore conceptual, not methodological. Cultural psychology views culture and psychology as mutually constitutive and treats basic psychological processes as culturally dependent, if not also, in certain cases, as culturally variable. Cross-cultural psychology, on the other hand, treats psychological processes as formed independently of culture, with cultural impacting on their display, but not on their basic way of functioning (Miller 2001, p. 38). In order to not remain too abstract, let us consider some pieces of research in contemporary cultural psychology.

According to contemporary cultural psychologists, working with a more nuanced and process-oriented understanding of culture, realizing and determining its impact on psychic functioning will broaden psychological theory. And indeed, with regard to a number of basic issues in psychology like cognition, emotion, the self, well-being, self-esteem, motivation, cultural psychological research has contributed to the elaboration of new theoretical frameworks (Kitayama and Cohen 2007). A core insight from the cognitive revolution has been that individuals in making sense of experience go beyond information given, rather than mere passively “processing” it (Bruner 1990). An act of interpretation mediates between stimulus and response. Such interpretation necessarily draws on culturally available systems of meaning. Culturally different settings require different activities, leading to different (cognitive) abilities. Thus, to refer to just one example, it was found that arithmetic problem-solving goes on differently, leading to different results, in different situations. Lave et al. (1984) found, for example, that whereas 98% of problems were correctly solved by subjects when engaged in grocery shopping, only 59% of an equal kind of questions were answered correctly by the same subjects when tested in a classroom. These researchers argue further that problem-solving is not a disembodied mental activity, but belongs to, and is specific to, the kind of situation the subject is involved in. In general, cognition is viewed as constituted, in part, by the concrete practical activities in which it is situated and the cultural tools on which it depends (Miller 1999, p. 87). Likewise, emotions are not just the same constant ones, differing only in degree across cultures, but are different in different cultures, i.e. some emotions exist in some cultures and not in other ones. Emotions are characterized by beliefs, judgments, and desires – the content of which is not natural but is determined by the systems of cultural belief, value and mores of particular communities. They are not natural responses elicited by natural features which a situation may possess, but socio-culturally determined patterns of experience and expression which are acquired, and subsequently feature in, specifically social situations (Armon-Jones 1986).

Also in the conceptions of the self – understood as an individual’s understanding and experience of the own psychic functioning – and in related modes of psychic functioning qualitative differences exist between individuals from cultural communities characterized by contrasting self-related cultural meanings and practices (Kitayama et al. 2007). Thus, researchers Shweder and Bourne (1984) showed that in person descriptions Oriyan Indians – compared to Euro-Americans – place greater emphasis on actions than on abstract traits, while more frequently making reference to the context. (Instead of describing a friend as, e.g., “friendly”, Oriyan Indians would say that she or he “brings cakes to my family on festival days.”) Recent extensions of this type of research indicate that theory of mind understanding does not spontaneously develop toward an endpoint of trait psychology, but that it proceeds in directions that reflect the contrasting epistemological assumptions of local cultural communities (Lillard 1998; Miller 2002). Another example: the fundamental attribution error (i.e. a bias to overemphasize dispositional relative to situational explanations of behavior) was formerly assumed to be universal, but research suggests that Asians may be less vulnerable to it than North Americans (Lee et al. 1996; Morris et al. 1995).

With regard to self-esteem and well-being, cultural research implies that strategies of self-enhancement and defensive self-promotion to maintain positive feelings about the self are culturally variable, with Japanese populations emphasizing a culturally supported self-critical stance and Chinese populations emphasizing maintaining harmony within groups. The tendencies for reported self-esteem and life satisfaction to be higher among North-American than among Asian cultural populations (Diener and Diener 1995) probably may not be viewed as indicating more successful patters of adaptation to be linked with individualism. Moreover, the research in this area suggests that psychological measures of self-esteem are biased by conceptions of norms, practices and self-conceptions as individualistic, and may therefore not be able to capture central goals for the self in cultures that emphasize fulfillment of interpersonal responsibilities and interdependence (Miller 2001, p. 33).

With regard to motivation, recent cultural work challenges common assumptions that link agency with individualism and shows that agency is experienced qualitatively different in contrasting cultural communities. In cultural groups where the self tends to be conceptualized as inherently social rather than as inherently autonomous, individuals are more prone to experience their true selves as expressed in the realization of social expectations rather than in acting autonomously. Also, Miller and Bersoff (1994) showed that whereas Americans interpret helping as more endogenously motivated and satisfying when individuals are acting autonomously rather than in response to social expectations, Indians regard helping in both cases as just as endogenously motivated and satisfying. Likewise, Iyengar and Lepper (1999) found that Euro-American children show less intrinsic motivation when choices on anagram and game tasks were made for them by their mothers or by their peer groups, but that Asian-American children also display highest levels when acting to fulfill the expectations of these trusted others. Extending this type of cultural research to issues of socialization, it has been shown that not only the meaning but also the adaptive consequences of particular modes of socialization are culturally dependent. Whereas in Euro-American cultural communities authoritarian modes of parenting tend to be associated with more maladaptive outcomes than are less controlling authoritative modes of parenting, Korean adolescents associate greater perceived parental warmth with greater perceived parental control, concordant with the Korean view of parents as having a responsibility to exercise authority over their children, with the failure to exercise it experienced as parental neglect (Berndt et al. 1993; Miller 2001).

## Turning to Religion as Object of Research

In psychology at large, this sensitivity for the cultural character of the phenomena under research has largely been lost: all too often, researchers take their results to be cross-culturally valid (there usually is no realization that results obtained [frequently only on Western middle class white students] may be only valid for the sample chosen, and even that only for the time being). Therefore, in spite of (or perhaps because of) dealing with small-scale questions, concepts and manipulated variables, and in spite of it ever increasing refinement of scales and sophisticated statistical techniques, psychology is being criticized for not observing sufficiently, not going deeply enough into the phenomena it wants to explore, especially not when constructing its “measuring instruments.” One of the main reasons for this lack of relevance is, according to Giorgi (1976), psychology’s problematic self-understanding. Because it chose to emulate the natural sciences it could not solve this fundamental dilemma: being faithful to the demands of the life-world and not doing justice to science, or remaining faithful to the requirements of science and precisely because of that, not doing justice to the life-world. From a cultural psychological viewpoint, it is especially deplored that psychology naturalizes its object of study, and that its *modus operandi* is marked by de-subjectivization and de-contextualization.

In the founding days of psychology and many other social sciences, however, many authors still had a clear interdisciplinary approach. A case in point is Wilhelm Wundt, one of the most influential of psychology’s founding fathers. According to him, psychology has two tools at its disposal: for investigations into the elements of consciousness (sensation, feelings, simple affects) the experiment should be employed, but for those psychic phenomena dependent upon certain historical-cultural conditions for their genesis and maintenance, what he called the “genetic method of cultural psychology” should be employed. Religion clearly being of the second kind of phenomena outlined by Wundt, he considered it to be an obvious subject for a cultural psychological approach, and consequently he devoted major sections of his outline for a cultural psychology to the subject of religion: of the ten volumes that his *Völkerpsychologie* comprises, three deal with religion (Wundt 1905/1920). Because almost all of his students were trained in experimental methods only, they usually adopted and developed only the first part of Wundt’s program (one of the reasons why the experiment is even by many contemporary psychologists regarded as the preferred, “really scientific” method in psychology). Religious phenomena are hardly suitable for experimental research; so where the experiment became dominant in psychology, the efforts to psychologically investigate religion usually declined. As Wundt’s cultural psychological program hardly found any followers, his way of doing psychology of religion never attracted many students. Most psychologists of the day concentrated on what he called “individual psychology”: on the investigation of psychic phenomena found with individuals or with small groups of individuals.

Among other interdisciplinary authors from the founding days of psychology and other social sciences were also scholars, who are now frequently remembered as founding fathers of sociology such as Max Weber (1904/1984) and Émile Durkheim (1912). Yet, although one finds interesting (cultural) psychological approaches with them, especially in their work on religion, they are hardly read by psychologists anymore. Awareness of the cultural character of the religious phenomena under study, was since the beginning of the twentieth century also found with a number of theologians, who had developed into historians of religions or into comparative scholars of religion (Andrae 1926, 1932; Van der Leeuw 1926, 1928, 1932; Söderblom 1908, 1916, 1939). Very frequently, such scholars turned to psychology for interpretative frameworks (cf. Sharpe 1986). But as psychology in general narrowed down its perspectives, it lost its attractiveness to comparative scholars of religion and to others who would otherwise have been interested in psychology. If at all, they oriented themselves to psychoanalytical psychology. The work of this group of scholars — not developing psychological theory themselves, but using psychological viewpoints within another discipline or enterprise — largely belongs to the second form of cultural psychology, and will be left out of consideration here.

In current cultural psychology, there is a return to the interdisciplinary approach from the former days (Jahoda 1993). As one of the social sciences, psychology is in need of close collaboration with, e.g., historians, sociologists and anthropologists. Accepting that culture is a major constituting and regulating force in people’s self-definition, conduct and experience also requires a different kind of research than is usual in mainstream psychology of religion. The particular religious “form of life” (Wittgenstein) the human being is embedded in, can then no longer be neglected in favor of searching for some presumably inherent and invariable psychic structures. On the contrary, it is necessary to study people *engaging* in their particular “form of life”, not to take them out of it, by submitting them to experiments, tests or questionnaires in the “laboratory.” Accordingly, researchers have to turn to participant observation, analysis of personal documents, interview, group-discussion and other ecologically valid techniques. Further, it becomes necessary to study not the isolated individual, but also the beliefs, values and rules that are prevalent in a particular cultural situation, together with the patterns of social relatedness and interaction that characterize that situation. In any case, it appears erroneous to try to study the “individual mind” as such. Psychology cannot fulfill this task without the aid of other cultural sciences.

## Examples of Contemporary Cultural Psychology and their Possible Application to Religion

In contemporary cultural psychology a variety of concepts and theories is employed, drawing from different strains of thoughts (Triandis 2007). As there is no space here to cover the range even approximately, let us take a brief look at just some of them, and see what (a) a concept like habitus means, what (b) narrative approaches stand for, and (c) what theories of “action” (or “activity”) have put forward. Finally, (d) also an example from psychoanalytic reasoning will be provided.

- The notion that psychological phenomena depend on practical activities has a long tradition, ranging from Marx and Engels, to Dewey and contemporary thinkers like Bourdieu. Religious people very often cannot explain on a cognitive level why they perform as they do, for example, in rituals. Most often they have no knowledge of the “official” rationales for certain conduct. Accordingly Roman Catholics cannot account for their behavior during Mass, nor can Buddhists for the reasons for experiencing grief as they do (Obeyesekere 1985). Yet people perform perfectly in accordance with the expectations of their religious (sub)culture, often with a competence and to an extent that a foreigner will never learn to manage. Religion regulates conduct, although this conduct cannot be conceived of as the conscious following of rules. People’s conduct — in the broadest sense, also including their perception, thinking, emotion, needs, etc. — is regulated according to a scheme or structure, that is not consciously known. This scheme is not even of a primarily cognitive nature at all, but is something belonging to the body. People act not because they know consciously what to do, it is as if their body knows for them. Affect, for example, is not the result of properly knowing how to feel — it is ruled by an immediate corporeal structure. Bourdieu (1980/1990) calls this structure *habitus*: it is this structure that generates and structures people’s actions. Although these structures are personally embodied, they are not individual: they characterize the (sub)culture and are derived from the patterns in the participant’s conduct. They belong to both the individual and a (sub)culture, in fact, they are precisely the nexus between an individual and a cultural institution. Unlike western secularized societies, religion in most cultures is not just a specific practice performed on specific occasions. In such cultures, religion is transmitted through practice, “without raising to the level of discourse. The child mimics other people’s actions rather than “models”. Body praxis speaks directly to motor function, in the form of a pattern of postures that is both individual and systematic, being bound up with a whole system of objects, and charged with a host of special meanings and values” (Bourdieu 1980/1990, pp. 73–74). The same applies to those western subcultures where religion still is predominantly a shaping and integrating force. For example: it is because he carries, in his body, the *habitus* of a Hindu from India, that a believer thinks, reacts, feels and behaves as an Indian Hindu, in fact *is* an Indian Hindu, and not because he would know the specifics of the doctrine, the ethical rules or the rituals. The believer usually is *not* aware of these specifics. Not being individual, the *habitus* is itself structured by social practices: its dispositions are durably inculcated by the possibilities and impossibilities, freedoms and necessities, opportunities and prohibitions inscribed in the objective conditions. It is in social practices that the *habitus* can be observed at work: being (re)produced and producing conduct itself.
- To what extent ever the *habitus* may be non-cognitive or operating in a way non- conscious to the actor, the conduct that results does mean something, both to the actor and to other cultural participants. This meaning is rooted in both personal life history and culturally available meanings. Analysis of activity must take into account the “forms of life” that are the context of meaning. This culturally available meaning can only be traced and analyzed at the level of text: words, proverbs, stories, myths and articulated symbols. However true it may be that without the analysis of activity, cultural psychology is only telling half of the story (Ratner 1996), it remains equally true that cultural knowledge, symbols, concepts and words, laid down in and maintained by linguistic conventions, stimulate and organize psychological phenomena. Here narrative psychology can be seen as an obvious ally in any analysis of religiosity. It points out that in the course of their life, people hear and assimilate stories which enable them to develop “schemes” which give direction to their experience and conduct — schemes with whose help they can then make sense out of a potential stimulation overload. To each developing story, and in every situation with which they are confronted, people bring an acquired catalogue of “plots” which is used to make sense out of the story or situation (Mancuso and Sarbin 1983). Here lies a possibility of applying narrative psychology to religious phenomena. For, whatever religion may be besides this, it is in any case also a reservoir of verbal elements, stories, interpretations, prescriptions and commandments which in their power to determine experience and conduct and in their legitimization possess narrative character. Clifford Geertz’s definition of religion, which is most widely disseminated in cultural psychology, points to the central importance of “stories”, of linguistically transmitted and given reality: “a religion is a system of symbols which act to establish powerful, pervasive and long-lasting moods and motivations in men by formulating conceptions of a general order of existence and clothing those conceptions with such an aura of factuality that the moods and motivations seem uniquely realistic” (1973, p. 90). In order to effect a connection with narrative psychology, one need only take the word “symbols” in this definition and give it more precise content with the aid of “stories and practices.” (In this connection one must realize that both practices and “conceptions” again employ stories to explain and legitimate themselves.) In other words: people who, among the various culturally available life forms, have also been introduced to, or have appropriated, a religious life form, have at their disposal a system of interpretation and conduct which (narratively) prefigures reality for them. Thus in every situation, expectations, interpretations and actions can be brought to bear which have been derived from a religious horizon of understanding and which, under certain circumstances, confirm and reinforce this understanding. Indeed, precisely those persons and groups are considered deeply devout who succeed, with the greatest frequency, spontaneously and perseveringly, to activate this religious horizon of understanding and who are in a position—despite the paradoxes they are confronted with—to overcome their own problems of religious interpretation and to act in harmony with the system of interpretation and conduct they have appropriated as well as with the “stories” that have been handed down to them.
- Activity theory was seminally lined out by the Russian psychologist Vygotsky (Veresov 1999). He enumerated three cultural factors that influence psychic functioning:

1. activities such as producing goods, raising children, educating the populace, devising and implementing laws, treating disease, playing, and producing art
2. artifacts including tools, books, paper, pottery, weapons, eating utensils, clocks, clothing, buildings, furniture, toys, and technology
3. concepts about things and people (e.g. the succession of forms that the content of person has taken in the life of human beings in different societies with their system of law, religion, customs, social structures, and mentality (Mauss 1938/1985, p. 3).

Vygotsky emphasized the dependence of psychic functioning on these three cultural factors, and the dominance of activities over the other two. (Ratner (2002) correctly pointed out that the real situation is more complex and dynamic: it contains reciprocal influence among the factors, and it is animated by intentionality, teleology, or agency.) Vygotsky stated: “The structures of higher mental functions represent a cast of collective social relations between people. These structures are nothing other than a transfer into the personality of an inward relation of a social order that constitutes the basis of the social structure of the human personality” (1998, pp. 169–170). Another member of the “cultural-historical” school in psychology initiated by Vygotsky wrote similarly that “changes take place in the course of historical development in the general character of men’s consciousness that are engendered by changes in their mode of life” (Leontiev 1981, p. 22).

According to activity theorists, activity, artifacts and cultural concepts need to be explored by psychologists to understand psychic functioning of individuals in a particular culture. This is not a task to be left to others than psychologists, as one has to look outside the individual to comprehend the content, mode of operation, and dynamics of psychological phenomena, constituted as they are by cultural factors and processes. Gerth and Mills (1953) pointed out that activities are internally divided into roles, and that each role entails distinctive rights, responsibilities, norms, opportunities, limitations, rewards and qualifications. (The activity of religion, e.g., includes the roles of believer and usually of some kind of priest, both more often than not divided into a host of religious categories as penitent, possessed, enlightened, etc., or such as pastor, baptizer, minister, exorcist, etc.) The distinctive characteristics of a role shape the occupant’s psychic functioning, for it is by her or his experience in enacting various roles, that the person incorporates certain objectives and values which steer and direct her or his conduct, as well as the elements of her or his psychic structure. Fulfilling a role requires psychic training: it involves learning what to do, as well as the meaning of what to do. “His [sic] memory, his sense of time and space, his perception, his motives, his conception of his self, his psychological functions are shaped and steered by the specific configuration of roles which he incorporates from his society” (Gerth and Mills 1953, p. 11; cf. also Ratner 2002, for a actualized outline of activity theory, integrating numerous contemporary research findings and extensive discussions of its relation to other cultural psychological approaches).

The concept of the (social) role is an excellent device for a cultural psychological approach to religion, as it designates a historically specific set of norms, rights, responsibilities and qualifications that pertain not only to actually present persons and/or situations, but also to those from the realm of religious stories, symbols and discourse in general. Roles are specific, distinctive ways of acting and interacting, and the concept can be used to designate the functioning (action, but also corresponding attitudes, emotions and expectations) on the part of the actual believer as well as to the (anticipated) conduct of the beings from an immaterial realm as stipulated by the divers religions, as the Swedish psychologist of religion Hjalmar Sundén (1959/1966) pointed out. His role theory of religious experience has proved a powerful heuristic device to analyze both contemporary and historical cases, and can be considered as a contribution to a cultural psychology of religion (Belzen 1996).

(d) In some psychoanalytic circles — notably in France and in those that orient themselves towards developments there — there is an awareness of the impact of culture, that seems contrary to much vulgarized psychoanalytic reasoning found so often. There is a recognition, that supra-individual entities like societies and/or entire cultures are not just repeating the phases and mechanisms that psychoanalytic theorizing claims to have discovered when studying patients. Instead, structurally informed analysts emphasize the importance of what Lacan called the “symbolic order” or the *discours de l’Autre*. This symbolic order preexists the individual and will persist when the individual has left it. Yet, the individual is already represented in this order before birth, even if only by the name that will be given. Lacan clearly gave primacy to cultural order when he invented his dictum: “man talks, yet because the symbol has made him man” (1966, p. 242). Psychic development is the result of culture, there is no natural—in the sense of innately preconceived—growth, according to Lacan. The structure of the psyche as such (not just it’s culturally variable contents) is dependent on culture, on forces from “outside”. The constitution of the subject, the “psychic birth” (after natural birth) is dependent on (awareness of the separateness of) “other” (usually the mother); in order to achieve a first (“imaginary”) image of itself, the child (in the so-called “mirror-phase”) needs someone else to pass down this image. Most important for cultural psychology: self-consciousness, in Lacan’s view, only emerges thanks to language: it is because of identification with the “discourse of the other” that the human being becomes a participant in culture, able to say “I” and, later, to speak in its own name. Subjectivity is constituted and marked by cultural givens. Because of the entrance into the cultural symbolic order (preeminently language), needs are transformed into desires, which are therefore not naturally given, but a product of culture. In this sense, it is impossible to conceive of a human instinct that would not be marked by cultural references that define it. Even sexual instincts are never merely natural forces: the strata of meanings deposited in them invariably condition the strategies of satisfaction as well as the pitfalls of suffering and discontents. That human beings desire, and the way in which they want to satisfy that desire, is the consequence of cultural signifiers that direct human desire. Thus, similarly as Freud defined the drive as psychic *labor* because of the intrinsic unity with the corporeal, also culture imposes labor, it shapes the psychic realm.

Again, modesty is essential here: cultural psychology is an indispensable part of the whole of psychology, but as any other part of psychology, it is limited. Cultural psychology is about some of the fundamentals of human psychic functioning, but not about all and it does not deny the importance of any of the others. All valid psychological approaches are valuable, and ideally they could all be integrated into a balanced theoretical method of the whole of psychology (a model that seems more beyond reach than ever before, to be honest).

## A Modest Stand

Cultural psychology provides a number of perspectives that come as a balance to some of the issues that have been haunting psychology of religion for a long time. As evoked in the beginning of this paper, very often psychologists have tried to anchor religion in some property of the human psyche. (For a very recent example, see Sperry 2012.) Yet, there is no need for assumptions about humans being religious by nature. Indeed, cultural psychology is more radical: it argues that there is no such thing as a human nature independent of culture. What is called religion certainly may be a very important cultural factor influencing individual human psychological functioning, and religiosity, religion’s correlate on an individual and social level, may display all kinds of psychological characteristics, but in the same way that law, sports, traffic, and what have you, are no individual creations, religion is not the result of individual human activity. No longer assuming that human being would necessary by nature, the effort to anchor religion in any special propensity of the human psyche turns out to be not only superfluous, but even erroneous: *pace* Piedmont (1999), there is no need to assume something like spirituality as sixth “fundamental factor of human personality” in order to explain the existence of religion. Also, the empirical finding that unbelievers may be mentally just as healthy as believers, or that believers may be just as disturbed as unbelievers, or that religion sometimes may be anything but beneficial to health and well-being need not come as spectacular or even be puzzling (as it has been and sometimes still is to a major fraction of clinical psychologists of religion).

Understandable enough, some psychologists of religion have taken pains to try to show that the discipline of psychology would be incomplete without paying attention to religion. If the human psyche would be religious by nature, it indeed would be logical to try to find the where and how of the psychological roots of religion. But after some 120 years of the existence of a field called psychology of religion, psychology at large has not been convinced of the adequacy of this position. Although some present colleagues authored papers with titles like “what the study of religion offers psychology” (Hill 1999), it is significant that they are published in media belonging to the psychology of religion, not in journals of general psychology. (In other words, such papers make the impression of preaching to the converted….) If we understand psychology of religion to be about religion, the urge to impress psychology at large disappears. The field’s aim should be to instruct anyone interested in religion, or rather, from a cultural psychological perspective, the aim should be to instruct about some of the psychological aspects of a certain phenomenon – be it an individual experience, an event, a state of affairs or anything else considered to be religious at a certain time and at a certain place. Religion does not belong to the formal object of psychology, it is not an entity like cognition, emotion, self and what have you, but it is one of the many possible material objects of psychology. Psychology’s contribution to the exploration and explanation of anything empirical is necessary, but limited: psychology is only about psychological aspects of anything under scrutiny. Therefore, psychology of religion needs to remain modest too: psychology is not called upon to explain the existence of religion *tout court*, but psychology may be drawn upon to explore the psychological dimensions of anything religious.

If the all too pretentious aspirations about psychology’s contribution to the study of religion are abandoned, some criticisms to the psychology of religion might be countered too. Very rapidly after its inception, observers of the psychology of religion have started to lament about the little that psychology of religion, despite all its efforts, would have to tell about religion. Around 1900, the expectations towards psychology in departments for religion or for theology were considerable: after the inception of history and forerunners of anthropology as intellectual tools to the study of religion, it was expected that psychology would be able to explain why the things assessed by other empirical discipline would the way they are. But after some decades, interest in psychology of religion faded away (Belzen 2015), and at the moment in the main only some interest in psychoanalysis and/or Jungian psychology is found there (see Parsons 2016). One of the criticisms of the psychology of religion all through the twentieth century has been: psychology of religion has its orientation with psychology, it tries to be fully acceptable there, but does not have much to say about what it is supposed to be about, namely religion (Koepp 1920; Nørager 1996b). To some extent, this criticism is understandable, but it seems also a bit unfair. For obviously, psychology of religion is psychology, it must be and continue to be a part of psychology, it must remain in touch with the mother-discipline and try to integrate the latest developments over there into its research on religion. But when psychologists of religion approach religion just as an example of what psychology in general has been finding out already, or if a religious person or population only serves as an additional potential sample to validate a psychological instrument or technique, the result is quite different from research that sets out with the primary aim to deal with a specific religious phenomenon, be it in a psychological way. Although ideally, the two should never be in contradiction with one another, there is a difference in emphasis between psychology of religion striving to be an element of modern psychology and a psychology of religion that wants to contribute to research of something religious. In the latter case, a broader treatment of the phenomenon is often included, its history and context are dealt with, in short: such research moves into the direction of interdisciplinary research, and by doing so it loses, in the gaze of many contemporary psychologists, its psychological focus.

To a psychology of religion aiming to focus on a specific religious phenomenon, cultural psychology comes as a natural ally, however. Cultural psychology of any specific phenomenon, religious or not, will try to thoroughly understand, if possible even “from within”; it will take time for extensive exploration and description, it will take seriously the accounts given by the persons themselves involved in or reporting on the phenomena. So, a cultural psychologist will not necessarily analyze her own experience. When it comes to anything religious, one does not need to practice the type of religion under scrutiny oneself, but one will probably cooperate with adherents of that particular religion, treating such representatives not as subjects in a standardized, preferably lab-like situation, but as partners in a search for psychological insight. (Very often, before external publication data and preliminary results will be discussed with such inside-experts with regard to the phenomenon studied.) As the illumination of the phenomenon is the primary aim, and not the reduction or modification to existing psychological methods or techniques, sometimes even the invention and development of new methods or techniques will be part of the investigation.

Granted, and to stay modest again, what from a cultural psychological perspective seems to be an advantage, may appear to colleagues with a different orientation to be a disadvantage: this type of empirical research takes considerable time, is very demanding towards the researchers (who may need to adapt to their partners in dialogue, requiring perhaps to spend considerable time with them, perhaps even to live with them, as in anthropological fieldwork) and is no guarantee at all for valid results. In a time where the pressure is high to turn out as many diploma’s in psychology with as little effort as possible, turning to standardized methods, easier to apply and quickly generating data to be analyzed by means of statistical software already available, is very understandable. But especially if one wants to bring somewhat exceptional (even within a given religious subculture) religious phenomena into the lens of research (think of stigmata with some Catholics, martyrdom with some Muslims, being slain in the spirits with Pentecostals, despair about eternal doom with some Calvinists – to give just a few examples religions dominant in the West) this approach proves to be more illuminative than sending out questionnaires or looking for changes in cerebral substratum. (Note, research into the psychophysiological underpinnings of any type of behavior, therefore also of religious behavior, is just as well possible, as it is possible into the psychophysiological underpinnings of swimming, reading or planning a holiday. But in order to understand why someone who always prayed to the Virgin Mary starts to pray to Allah, or perhaps discontinues to pray at all, or to even understand why such prayer can have become so important for an adult that she or he would rather die than discontinue practicing it, psychophysiological knowledge is not of prime relevance. After all, brain activity in prayer to Allah or to Mary is not different, but to praying persons the question to whom to pray makes all the difference.) As already Aristoteles pointed out, scientific approach, method and technique should be appropriate to the object of research, but they also depend on the kind of questions one asks – and about the same phenomenon many different questions can be asked, from a great number of different scientific disciplines. A bit more radical, one could even say that there are no empirical phenomena that can be restricted to one discipline only; even if we take “typical” psychological phenomena, dreaming, cognition, emotion or whatever, it would be silly to neglect that they can approached from disciplines like physiology, history, anthropology, etc.

The quite demanding and usually time-consuming character of empirical cultural psychological research may help to explain there is no abundance in reporting of this type of psychological research (Gillespie and Zittoun 2015). On the contrary, there is a clear tendency among cultural psychologists to be stronger in theory than in empirical research: Contemporary authors like Ratner, Straub or Valsiner publish much more theoretical than empirical work (Ratner 1991, 2002, 2006, 2008, 2012; Straub 1989, 1999; Straub and Weidemann 2015; Valsiner 2007, 2012, 2014; Valsiner and van der Veer 2000).

What counts as progress or as an advantage to one psychologist, may be seen as a disadvantage to others. As pointed out, cultural psychologists will try to keep away from constructing a concept of religion all of their own; for theoretical orientation and instruction, they will turn to other disciplines, and with regard to empirical research, they will accept what research participants tell them to be religion. (Therefore, cultural psychologists will also try to grant no priority to any form of religion. Obviously, this stand will not solve all problems about the definition and conceptualization of religion, yet the striving to solve such problems by way of an all embracing definition is suspect to researchers in this tradition.) The heterogeneous character of the results achieved at in this way is acknowledged, and not tolerated as a first step towards overarching theory, but is rather accepted to reflect the non-cumulative character of knowledge within the field called psychology, especially when it comes the heterogeneous multitude of phenomena called religious. Mind, however, that such a conclusion is not specific to cultural psychologists, but is also reached by proponents of a nomothetic-quantitative orientation in the psychology of religion (cf., e.g. Spilka et al. 1985; Spilka and Ladd 2013). It is the evaluation that is different, not so much the assessment of the current state of affairs in the psychology.

To sum up: There are strengths and limits to cultural psychology. It does not claim at all to be the only approach valuable if a psychologist want to do research on religion. It is only one possible approach, composite in itself and drawing on a variety of theories, insights, methods and techniques, but working on one of the fundamental aspects of human psychological functioning, it is as indispensable to efforts to explore and understand anything called religious as any other psychological approach may be. Depending on the type of questions one strives to answer about a particular religious phenomenon, it may sometimes be more, sometimes be less appropriate than other types of psychology. Whether researchers will employ cultural psychology or another approach from contemporary psychological sciences will depend on their personal preferences, their professional training, the type of context they are functioning in and hopefully also on the kind of phenomenon pointed out to them as religious by a certain (sub)culture.

## Footnotes

1 When C. Daniel Batson some decades later expanded this typology by empirically assessing a third type (“quest-religiosity”), this type proved to be a fair rendering of his own religiosity (Batson and Ventis 1982). According to the contemporary psychologist Jesse Bering, a confessing atheist, psychology explains why religious belief is wrong (Bering 2010). Yet according to his antipode, the evangelical Christian and “cognitive scientist of religion” Justin Barrett, psychology shows that being religious is the default setting of the human being, as it is the result of our divine creation (Barrett 2011). The examples of psychologists apparently confirming by means of “empirical research” what their religious apriori’s already told them, are abundant.

2 An exception here is the recent two-volume handbook published by the American Psychological Association: it explicitly tries to call for an “applied” psychology of religion (Pargament 2013).

3 Cultural psychologists usually define meaningful action or conduct as the object of psychology. Obviously, there are also forms of human behavior that are not intentional or not regulated by meaning (like drawing back one’s hand from a hot object; although even in the way this is done, there exists cultural variation).

4 Recently, authors from both traditions are trying to open a dialogue and to look for commonalities instead of stressing differences (cf. e.g. Kitayama and Cohen 2007; Matsumoto 1994a, b, 1996; Valsiner and Rosa 2007).
